# Ear Disease and "Head Symptoms" in Infants

**Published:** 1897-10-30

**Authors:** 


### EAR DISEASE AND "HEAD SYMPTOMS" IN INFANTS.

It would be difficult to exaggerate the importance of the part taken by the ear in the production of the "brain symptoms" which occur in the course of many infantile disorders. Very often, indeed, the symptoms pointing to the brain seem to stand by themselves and form the main feature of the illness, and unless the possibility of their arising from the ear be borne in mind there is risk, on the one hand, of their being looked upon as indicating meningitis, or, on the other hand, of their being treated as if they arose from "reflex irritation."

We are not concerned to deny what is among the first articles of faith with many practitioners, viz., that convulsions, retraction of the head, and other cerebral symptoms are often due to reflex irritation, and cease, for example, on the expulsion of intestinal worms. It is well, however, to remember that, in the ordinary methods adopted for the removal of these entozoa, a clean sweep is made of many toxic substances lodging in the intestines, and that in such cases it is far more probable that the cerebral symptoms have been due to a toxæmia than to any mere reflex irritation set up by such worms as may prove to have been present.

Whatever may be the efficacy of reflex irritation as a producer of disease in certain cases, there can be no doubt that a too easy belief in it as an all-explaining cause of infantile complaints is the origin of much feeble diagnosis and inefficient treatment.

In the investigation of "brain symptoms" in children, then, the very first thing to do is to discover or exclude the presence of gross physical disease—often by no means an easy task; and among such disorders those of the ear claim the first attention, partly because of all routes to the brain that by the ear is the most vulnerable, and partly because, in consequence of the great frequency of pharyngeal diseases in children, the route by the eustachian tube and ear is the one most frequently attacked.

The ear, moreover, is important in regard to cerebral symptoms, not only as being the main route by which infective disorders reach the brain, but also because its diseases may accurately simulate those of the brain itself.

It may, for example, be quite impossible to distinguish between early basic meningitis and otitis media. Thus we may have marked head retraction, fever, extreme irritability, vomiting, and even convulsions—all symptoms compatible with and highly characteristic of commencing posterior basic meningitis—and yet find them relieved by incision of the tympanic membranes, and subsequent escape of thin muco-purulent fluid, and complete recovery may take place.

In a careful *résumé* in the *Practitioner* of what has been written on the subject, it is shown that, although after incision of the membrana tympani and the use of the Politzer bag a good result often occurs, in other less fortunate cases there has been only temporary relief from the operation, the further progress being marked by renewed cervical opisthotonos, tonic spasm of the limbs, cerebral effusion, and death, leaving post-mortem evidence of meningitis of the posterior base.

Clearly the cases which have been relieved by incision, and also those which are relieved by a fortunate rupture of the membrane, must be looked upon as having escaped not merely from an otitis media, but from a disease which, if left alone, might have become a meningitis. The problem, so far as regards treatment, is put in the following way by Dr. Barlow: "In a given case there is a chance that incision of the tympanum may give relief, and eventuate in perfect recovery, and, if it does not procure more than temporary relief, the operation, performed by a competent surgeon, does no harm."

Dr. Lees points out that, owing to the incomplete development of the petrous bone during the first year of life, otitis is very apt to infect the cerebral meninges, and that this extension may occur when symptoms pointing definitely to the ear have been slight or absent. Retraction of the head is the most important symptom, and when this occurs within a few days after the commencement of the illness, especially if it is accompanied by vomiting or any kind of convulsion, the question of paracentesis should always be considered at once. Spontaneous discharge from the ear must never be waited for. But all this is ancient history. The difficulty is to get the knowledge we possess brought into general use. Sir William Dalby, writing recently on the subject, pointed out that the advisability of incising the tympanic membrane was insisted on by Toynbee, and has long since become an ordinary routine among aural surgeons. As he states, however, the real difficulty in these cases is to decide upon the precise period when the incision should be made, for even the appearance of the tympanum itself may give but little clue to the condition. "The constitutional symptoms and the demeanour of the little patient, as indicating pain of an acute and agonising character . . . are the sole evidences within our hands."

Where leeches and hot fomentations fail to cut short the disease (and if they do relieve they relieve at once), it would, he says, be safe to adopt the rule of making a vertical incision in the posterior section of the membrane.

Some assistance in diagnosis may be gained from a consideration of the following signs, which may, when they occur, be taken as suggesting the ear as the cause of the illness: The child constantly endeavours to rub the affected organ; it utters a sharp cry of pain when pressure is made below the meatus; and it refuses to rest its head on the affected side.

